# Long Non-Coding RNA MEG8 Suppresses Hypoxia-Induced Excessive Proliferation, Migration and Inflammation of Vascular Smooth Muscle Cells by Regulation of the miR-195-5p/RECK Axis

**DOI:** 10.3389/fmolb.2021.697273

**Published:** 2021-11-01

**Authors:** Dexing Xu, Ruozhu Dai, Hao Chi, Wen Ge, Jingfeng Rong

**Affiliations:** ^1^ Department of Cardiology, The First Hospital of Quanzhou Affiliated to Fujian Medical University, Quanzhou, China; ^ **2** ^ Department of Cardiothoracic Surgery, Shuguang Hospital Affiliated to Shanghai University of Traditional Chinese Medicine, Shanghai, China; ^3^ Department of Cardiology, Shuguang Hospital Affiliated to Shanghai University of Traditional Chinese Medicine, Shanghai, China

**Keywords:** vascular smooth muscle cells, hypoxia, MEG8, MiR-195-5p, RECK, hypoxia inducible factor 1 subunit alpha

## Abstract

It has been recognized that rebalancing the abnormal proliferation and migration of vascular smooth muscle cells (VSMCs) helps relieve vascular injury. Presently, we aim to investigate whether long non-coding RNA (lncRNA) maternally expressed 8 (MEG8) plays a role in affecting the excessive proliferation and migration of VSMCs following hypoxia stimulation. A percutaneous transluminal angioplasty balloon dilatation catheter was adopted to establish vascular intimal injury, the levels of MEG8 and miR-195-5p in the carotid artery were tested by quantitative reverse transcription-polymerase chain reaction (qRT-PCR). Hypoxia was used to stimulate VSMCs, then the cell counting kit-8 (CCK-8) assay, Transnwell assay, and wound healing assay were conducted to evaluate the proliferation, and migration of VSMCs. The protein levels of RECK (reversion inducing cysteine rich protein with kazal motifs), MMP (matrix metalloproteinase) 3/9/13, COX2 (cytochrome c oxidase subunit II), macrophage inflammatory protein (MIP)-1beta, VCAM-1 (vascular cell adhesion molecule 1), ICAM-1 (intercellular adhesion molecule 1), and HIF-1α (hypoxia inducible factor 1 subunit alpha) were determined by western blot or cellular immunofluorescence. As the data showed, MEG8 was down-regulated in the carotid artery after balloon injury in rats and hypoxia-treated VSMCs, and miR-195-5p was overexpressed. Forced MEG8 overexpression or inhibiting miR-195-5p attenuated hypoxia-promoted cell proliferation and migration of VSMCs. In addition, miR-195-5p up-regulation reversed MEG8-mediated effects. Hypoxia hindered the RECK expression while boosted MMP3/9/13 levels, and the effect was markedly reversed with MEG8 up-regulation or miR-195-5p down-regulation. Mechanistically, MEG8 functioned as a competitive endogenous (ceRNA) by sponging miR-195-5p which targeted RECK. Moreover, the HIF-1α inhibitor PX478 prevented hypoxia-induced proliferation, and migration of VSMCs, upregulated MEG8, and restrained miR-195-5p expression. Overall, lncRNA MEG8 participated in hypoxia-induced excessive proliferation, inflammation and migration of VSMCs through the miR-195-5p/RECK axis.

## Introduction

Vascular smooth muscle cells (VSMCs) are the main component of the vascular wall, which are crucial for maintaining intravascular pressure and tissue perfusion. Vascular endothelium protects VSMCs from blood stimulation under normal physiological conditions, but VSMCs are abnormally differentiated after mechanical injury or contact with blood growth factors (Wang et al., 2015). Unbalanced VSMCs’ proliferation, migration, and apoptosis are drivers of multiple vascular diseases, such as hypertension, atherosclerosis, and pulmonary hypertension (Tykocki et al., 2017; Morris et al., 2019). Therefore, it is urgent to study the specific mechanism of VSMCs in vascular lesions.

Long non-coding RNAs (lncRNAs) are noted as non-protein-coding transcripts which are longer than 200 nucleotides. Abnormal expression of lncRNAs plays an important role in diversified physiological and pathological processes of cells, including endothelial cells and VSMCs (Lim et al., 2020; Daidone et al., 2021). For example, silencing small nucleolar RNA host gene 14 (SNHG14) suppresses LPS (lipopolysaccharide)-induced apoptosis and inflammation of human lung epithelial cells (Zhu et al., 2021). Hu et al. showed that Metformin enhanced the AMP-activated protein kinase (AMPK) activity and hampered phenotypic conversion of VSMCs by up-regulating antisense non-coding RNA in the INK4 locus (ANRIL), thus preventing the development of atherosclerotic plaques (Hu et al., 2020). LncRNA MEG8 is located at 14q32.2-q32.31 on the chromosome, and it acts as an oncogene in cancer (Terashima et al., 2018; Guo et al., 2021). Other studies have found that down-regulation of MEG8 hampers oxygen-glucose deprivation-induced endothelial cell viability, migration and angiogenesis in mouse brain microvessels by targeting the miR-130a-5p/vascular endothelial growth factor A (VEGFA) axis, which alleviates cerebral ischemia after stroke (Sui et al., 2020). However, the relationship between MEG8 and VSMC differentiation was less understood.

MicroRNAs (miRNAs) are small endogenous molecules of 20–25 nucleotides in length that are involved in post-transcriptional regulation and epigenetic regulation of genes. Therefore, miRNAs play a crucial role in multiple diseases. Ye et al. found that up-regulating fibronectin type III domain containing 5 (FNDC5) via miR-135a-5p inhibition substantially weakens the proliferation and vascular remodeling of VSMCs and alleviates hypertension in spontaneously hypertensive rats (Ye et al., 2021). Xiao et al. showed that miR-22 targeted p38 mitogen-activated protein kinase (MAPK)α to restrain VSMC apoptosis in aortic dissection, indicating that miR-22 is a potential target for the treatment of aortic dissection (Xiao et al., 2020). As a member of the miR-15 family, miR-195-5p has a wide range of roles. As an example, up-regulation of miR-195-5p targets Yes-associated protein 1 (YAP1) to exert a tumor-suppressive role in cervical cancer (Liu et al., 2020). Other studies have substantiated that miR-195-5p is overexpressed in lung tissues of sepsis rats, and the up-regulation of cancer susceptibility candidate 9 (CASC9) improves sepsis-induced acute lung injury (ALI) by inactivating the miR-195-5p/PDK4 axis (Wang et al., 2020a). However, there are few studies on the effect of miR-195-5p on VSMCs.

Reversion-inducing cysteine-rich protein with Kazal motifs (RECK), located on chromosome 9p13.3, is a membrane-anchored glycoprotein. With the deepening of research in recent years, RECK has been found to interfere with the progression of diversified diseases. For example, LINC01419 targets enhancer of zeste homolog 2 (EZH2) to hinder the expression of RECK and heighten cell proliferation, migration and invasion in hepatocellular carcinoma (Zhang et al., 2020). In addition, RECK is a key regulator of extracellular matrix (ECM) and angiogenesis (Oh et al., 2001). Also, minocycline declines MMP2 and MMP9 activation by up-regulating RECK, thus reducing BB homodimer of recombinant platelet-derived growth factor (PDGF-BB)-induced VSMC proliferation and migration (Higashi et al., 2019). Therefore, we probed whether RECK can interfere with the biological process of VSMCs.

In brief, non-coding RNAs (lncRNAs and miRNAs) mediate the proliferation and migration of VSMCs. In this study, we found that MEG8 was down-regulated in hypoxia-induced VSMCs, whereas miR-195-5p was promoted by hypoxia stimulation. Functional assays were performed to investigate the MEG8-miR-195-5p axis in hypoxia-induced dysfunction of VSMCs. We found that overexpression of MEG8 regulated the miR-195-5p/RECK axis to curb the proliferation and migration of VSMCs. All over, we hope this study helps the understanding of hypoxia-induced dysfunction of VSMCs in vascular diseases.

## Materials and Methods

### Rat Carotid Artery Balloon Injury Model

Sprague-Dawley (SD) rats (30 rats, 280–320 g, 8–12 weeks) were purchased from the Animal Laboratory Center of Shanghai Jiao Tong University. Rats were fed in the SPF chamber for 1 week before the experiment. The carotid artery balloon injury model was constructed on rats by referring to the previous study (Tsaousi et al., 2011). In a nutshell, the rats were intraperitoneally injected with pentobarbital (40 mg/kg). After successful anesthesia, an incision was made in the middle of the neck of the rats to expose and dissociate the left common carotid artery, as well as the left internal and external carotid artery. Low molecular weight heparin (100 U/kg) was injected into the tail vein of rats for anticoagulation, and then the proximal end of the common carotid artery and the distal end of the internal carotid artery were clipped. A small incision in the distal end of the internal carotid artery was cut with ophthalmic scissors, and a balloon catheter was placed to the common carotid artery. The catheter was inflated with a syringe. When the balloon was full, it was turned back and forth three times to achieve damage to the intima and then deflated. On days 3, 7, 14, and 28 after endovascular injury, the left ventricle of rats was perfused with 4% paraformaldehyde. Then, the rats were euthanized, and carotid artery specimens were collected. The carotid artery balloon injury was not performed in the control group, and other procedures were the same as those in the experiment group. Four weeks after vascular intimal injury, hematoxylin-eosin (H&E) staining was applied for evaluating the histopathological changes of the internal carotid artery of the rats. All animal procedures were approved by the Animal Research Committee of The First Hospital of Quanzhou Affiliated to Fujian Medical University (Approve number: FMU-QZH-2021-020).

### Cell Culture and Hypoxia Stimulation

Rat aortic smooth muscle cells (A-10) were bought from American Type Culture Collection (ATCC, Rockville, MD, United States), and Human VSMCs (hVSMCs) were purchased from BeNa Culture Collection (BNCC; Beijing, China). The two cell lines were cultured in the Dulbecco’s modified Eagle’s medium (DMEM) (Thermo Fisher Scientific, MA, United States) comprising 10% fetal bovine serum (FBS, Gibco, LifeTechnologies, United States) and 1% penicillin/streptomycin (Invitrogen, CA, United States). The medium was placed at 37°C in an incubator containing 5% CO_2_. During the logarithmic growth phase, 0.25% trypsin (Thermo Fisher HyClone, UT, United States) was employed for cell passage. For hypoxia treatment, the cells were exposed to a mixture of hypoxic gases containing 1% O_2_, 5% CO_2_, and 94% N_2_ for 4 min and then incubated in a Seed Modular Incubator Chamber (Billups-Rothenberg Inc., San Diego, CA, United States) for 3, 6, 12, and 24 h. The HIF-1α inhibitor PX478 (25 μM, Cat.No. HY-10231, MedChemExpress, NJ, United States) was used for inhibiting HIF-1α.

### Cell Transfection

The hypoxia-induced A-10 cells and hVSMCs were taken and transfected with the overexpression plasmids of MEG8 and RECK, miR-195-5p mimics, and their corresponding negative controls (Ribobio, Guangzhou, China) using Lipofectamine-2000 (Invitrogen, CA, United States). The cells were cultured at 37°C and incubated with 5% CO_2_. Twenty-four hours after the transfection, the total cellular RNA was extracted for quantitative reverse transcription-polymerase chain reaction (qRT-PCR) to verify the altered miRNA expression in the transfected cells.

### Quantitative Reverse Transcription-Polymerase Chain Reaction

Total RNA was extracted using the TRIzol reagent (Invitrogen, Carlsbad, CA, United States) according to the experimental requirements. Afterward, NanoDrop™ 1000 Spectrophotometer (Thermo Fisher Scientific) was adopted to check the RNA purity and concentration. The absorbance ratio of OD260/OD280 was between 1.8 and 2.0. For RNA quality assessment, the concentration of extracted total RNA was between 1.2 and 1.6 μg/μl. The total RNA was reversely transcribed into cDNA by utilizing the Toyobo reverse transcription Kit (Toyobo, Osaka, Japan) and One Step PrimeScript miRNA cDNA Synthesis Kit (Bao Biological Engineering (Dalian) Co., Ltd.). qRT-PCR was performed on the ABI7500 real-time PCR system (Applied Biosystems, SanFrancisco, CA, United States) using SYBR Green qPCR Master Mix (MedChemExpress, NJ, United States). The cycling conditions were 95°C for 10 min, followed by 40 cycles of 95°C for 15 s and 60°C for 30 s. Expression levels were calculated using the 2^−ΔΔCt^ method. Glyceraldehyde-3-Phosphate Dehydrogenase (GAPDH) served as the housekeeping gene of lncRNAs, and U6 served as that of miRNAs. The Primer4.0 software was employed to design primers according to the sequence number of each gene in the GenBank. The primers used in this study are exhibited in Table 1. The experiment was repeated three times, and each time was performed in triplicate.

**TABLE 1:** Primers used in this study.

Genes	Forward (5′→3′)	Reverse (5′→3′)
MEG8-rat	cca​ggt​tca​agg​tcc​ctc​at	tct​tgt​gtc​tcg​aag​gcc​tt
MEG8-human	cttgcttcctggcacgag	caggaaacagctatgac
miR-195-5p-rat	ggg​gta​gca​gca​cag​aaa​t	tccagtgcgtgtcgtgga
miR-195-5p-human	ctg​gag​cag​cac​agc​caa​ta	agc​ttc​cct​ggc​tct​agc​a
RECK-rat	caa​gac​ggt​gac​caa​gca​aa	tcc​tcc​tgt​aag​tgg​cca​tg
RECK-human	gct​att​gcc​ttg​gag​tgt​cg	taa​ctg​caa​caa​acc​gag​cc
HIF-1α-rat	gaa​cgt​tac​tgc​agc​aac​ca	ttc​tgc​tgc​ctt​gta​tgg​ga
HIF-1α-human	cag​tcg​aca​cag​cct​gga​ta	cca​cct​ctt​ttg​gca​agc​at
GAPDH-rat	tgg​ggc​caa​aag​ggt​cat​ca	gca​gga​tgc​att​gct​gac​aa
GAPDH-human	cga​cca​ctt​tgt​caa​gct​ca	agg​gga​gat​tca​gtg​tgg​tg
U6-rat	ctc​gct​tcg​gca​gca​cat​ata​cta	acg​aat​ttg​cgt​gtc​atc​ctt​gcg
U6-human	ctcgcttcggcagcaca	aac​gct​tca​cga​att​tgc​gt

### Cell Counting Kit-8 Assay

Cell viability was evaluated by the CCK-8 assay. The stable transfected A-10 cells and hVSMC were inoculated into 96-well plates (2 × 10^3^ cells/well) and incubated at 37°C for 24, 48, 72 and 96 h, respectively, based on the experimental purposes. Then, 10 μl CCK-8 (Dojindo Molecular Technologies, Kumamoto, Japan) reagent was added to each well according to the manufacturer’s instructions. After incubation for 1 hour, the optical density (OD, 450 nm) value was measured on a spectrophotometer (Shjingmi Co., Ltd.). The experiment was repeated three times, and each time was performed in triplicate.

### Transwell Assay

Transwell assay was adopted for evaluating cell migration. The stably transfected cells were resuspended with the DMEM medium, and the cell density was adjusted to 1 × 10^5^/ml. 100 μl cell suspension was added to the upper chamber of the Transwell, and 500 μl DMEM complete medium was added to the lower compartment. After incubation at 37°C with 5% CO_2_ for 24 h. The cells that failed to migrate were wiped off, and the migrated cells were fastened with 4% paraformaldehyde and stained with crystal violet for 5 min. The number of cells entering the lower chamber was observed using a CX41 light microscope (×200, Olympus Corporation, Japan). The experiment was repeated three times, and each time was performed in triplicate.

### Wound Healing Assay

The stably transfected cells were spread in 6-well plates at 1 × 10^6^/ml. When the cells’ fusion rate reached 90–100%, the cells were scratched in the middle of the monolayer with a 1 ml sterile pipet. The floating cells were washed and removed by PBS and then cultured in the DMEM medium for 0 and 24 h, followed by fixation with 4% paraformaldehyde. Under an inverted microscope (Zeiss, Oberkochen, Germany), cell migration was assessed. The cell migration abilities were calculated as D24/D0, where D0 is the wound area at the initial time point, and D24 is the wound area at the observation time point (24 h). The experiment was repeated three times, and each time was performed in triplicate.

### Western Blot

The protein concentration was determined using a Bicinchoninic Acid (BCA) kit (Pierce, Rockford, IL, United States). The total protein was separated by 10% SDS-PAGE electrophoresis. After 2 h of electrophoresis, the protein was transferred to PVDF membranes (Amersham, Buckinghamshire, United Kingdom), which were sealed with 5% skimmed milk powder for 1 hour and incubated with the Anti-MMP3 antibody (1:1,000, ab52915, Abcam), Anti-MMP9 antibody (1:1,000, ab76003, Abcam), Anti-MMP13 antibody (NBP2-45887, 1:1,000, Novus, Biologicals, CO, United States), Anti-RECK antibody (Cell Signaling Technology, CST, Danvers, MA, United States), Anti-COX2 antibody (1:1,000, ab179800, Abcam), Anti-MIP-1β antibody (1:1,000, ab45690, Abcam), Anti- VCAM-1 antibody (1:1,000, ab134047, Abcam), Anti-ICAM-1 antibody (1:1,000, ab171123, Abcam), Anti-HIF-1α antibody (1:1,000, ab179483, Abcam), Anti-β-actin antibody (1:1,000, ab179467, Abcam), and Anti-GAPDH antibody (1:1,000, ab9485, Abcam) overnight. The following day, the membranes were rinsed with TBST. Horseradish peroxidase-labeled secondary anti-Goat anti-Rabbit IgG (1:3,000, ab150077, Abcam) was added and incubated at 37°C for 1 hour. The images were developed and preserved by ECL (Beyotime, Shanghai, China). The experiment was repeated three times, and each time was performed in triplicate.

### Dual-Luciferase Reporter Assay

By querying the Starbase (http://starbase.sysu.edu.cn/), we predicted the binding sites between miR-195-5p and MEG8, as well as between RECK and miR-195-5p. Accordingly, the wild-type (WT, containing the binding sites with miR-195-5p) or mutant (MUT, mutant for the binding sites with miR-195-5p) MEG8 or RECK vectors were synthesized by GENEWIZ (Suzhou, China). After that, 30 ng of MEG8-WT or MEG8-MUT and RECK-WT or RECK-MT plasmids were co-transfected with 50 nM of miR-195-5p mimics or negative controls into the cells. After 48 h, the luciferase activity was measured using a dual-luciferase assay kit (Promega, Madison, WI, United States). The experiment was repeated three times, and each time was performed in triplicate.

### RNA Immunoprecipitation

A RIP Kit (Millipore, United States) was applied to verify the association between MEG8 and miR-195-5p, as well as between miR-195-5p and RECK. In this study, cells were incubated with protein A/G Sepharose beads at 4°C in RIP lysate solution (Beyotime Biotechnology Co., Ltd., Shanghai, China), with IgG as a negative control. The experiment was repeated three times, and each time was performed in triplicate.

### Fluorescence *In Situ* Hybridization

A-10 cells were immobilized in 4% paraformaldehyde for 15 min, washed with PBS and transfused in 0.5% Triton X-100 at 4°C for 15 min, and digested with protease K for 5 min. They were then incubated with Cy3-labeled MEG8 and FITC-labeled miR-195-5p probes (GenePharma Technology, Shanghai, China) according to the manufacturer’s instructions. After staining the cells with DAPI, the fluorescence signals were observed under a fluorescence microscope (Zeiss, New York, NY, United States).

### Cellular Immunofluorescence

A-10 cells and hVSMCs were immobilized with 4% formaldehyde solution for 10 min, followed by Triton X-100 permeation for 10 min and 5% BSA blocking. The primary anti-RECK antibody (Cell Signaling Technology, CST, Danvers, MA, United States) was added and incubated overnight at 4°C, followed by secondary anti-Goat anti-Rabbit IgG (1:3,000, ab150077, Abcam) incubation at room temperature for 1 hour. Cells were subjected to 4',6-diamidino-2-phenylindole (DAPI, Beyotime, Shanghai, China) staining for 3 min, blocked and then observed by fluorescence microscopy. The experiment was repeated three times, and each time was performed in triplicate.

### Statistical Analysis

Data in this study were processed by the GraphPad Prism 8 statistical software (GraphPad Software, Inc., United States). The measurement data were expressed as mean ± standard deviation (x ± SD), and the data among the multiple groups were analyzed using one-way ANOVA followed with Tukey *Post Hoc* Test, while *t* test was used for analyzing two-group data. Value *p* < 0.05 was considered significant.

## Results

### Maternally Expressed 8 was Lowly Expressed in the Carotid Artery of Rats With Balloon Injury

**FIGURE 1:**
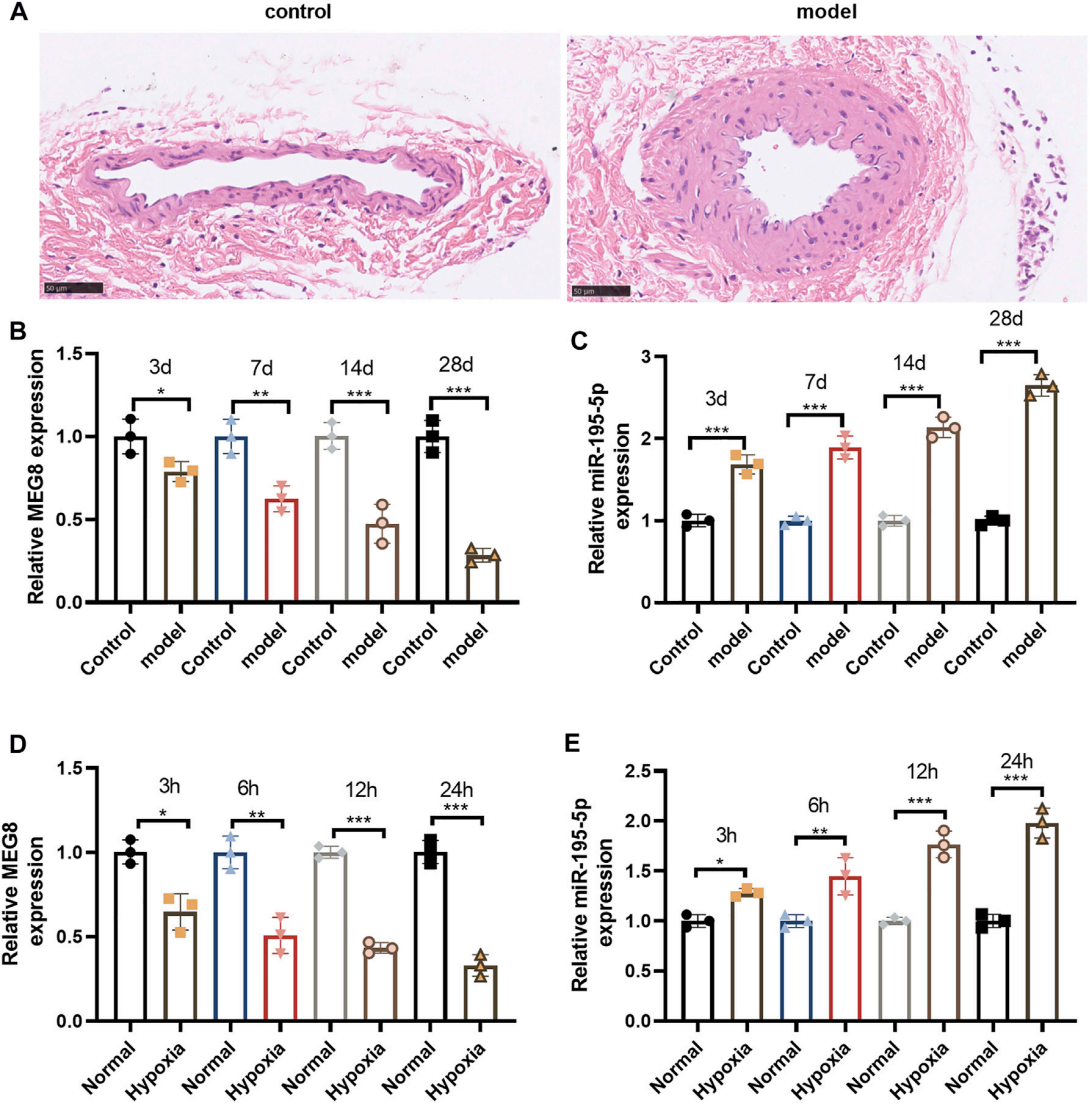
MEG8 was lowly expressed in the carotid artery of rats with balloon injury. The carotid artery injury model of SD rats was constructed by a percutaneous transluminal angioplasty balloon dilatation catheter. **(A)** H&E staining for detecting the histopathological changes of internal carotid 4 weeks after vascular intimal injury. **(B,C)** qRT-PCR checked the expression of MEG8 and miR-195-5p in the carotid artery of rats. **(C,D)** qRT-PCR was implemented to gauge the expression of MEG8 and miR-195-5p in A-10 cell lines treated with hypoxia. *, **, *** represents *p* < 0.5, *p* < 0.01 and *p* < 0.001 vs. control group or Normal group. Five rats were in each group. Each assay was performed in triplicate.

The carotid artery injury model was set up in SD rats. H&E staining results manifested that compared with that of the self-contralateral noninjured artery (control), the neointimal layer of the injured intimal artery (model) was markedly thickened, and the area of the vascular cavity was notably reduced (Figure 1A). qRT-PCR displayed that MEG8 was significantly down-regulated in the carotid artery of rats after balloon injury, and its expression decreased with the time of injury. However, miR-195-5p was significantly up-regulated time-dependently (*p* < 0.05, Figures 1B,C). Then, a hypoxic intervention was performed on A-10 cells. qRT-PCR results disclosed that the expression of MEG8 was curbed with the increase of hypoxic intervention time, while the expression of miR-195-5p was completely opposite (*p* < 0.05, Figures 1D,E). The above detection results suggested that MEG8 was significantly up-regulated and miR-195-5p was down-regulated in VSMCs with carotid artery injury and hypoxia.

### Overexpression of Maternally Expressed 8 Attenuated the Hypoxia-Induced Adverse Biological Behaviors of Vascular Smooth Muscle Cells

**FIGURE 2:**
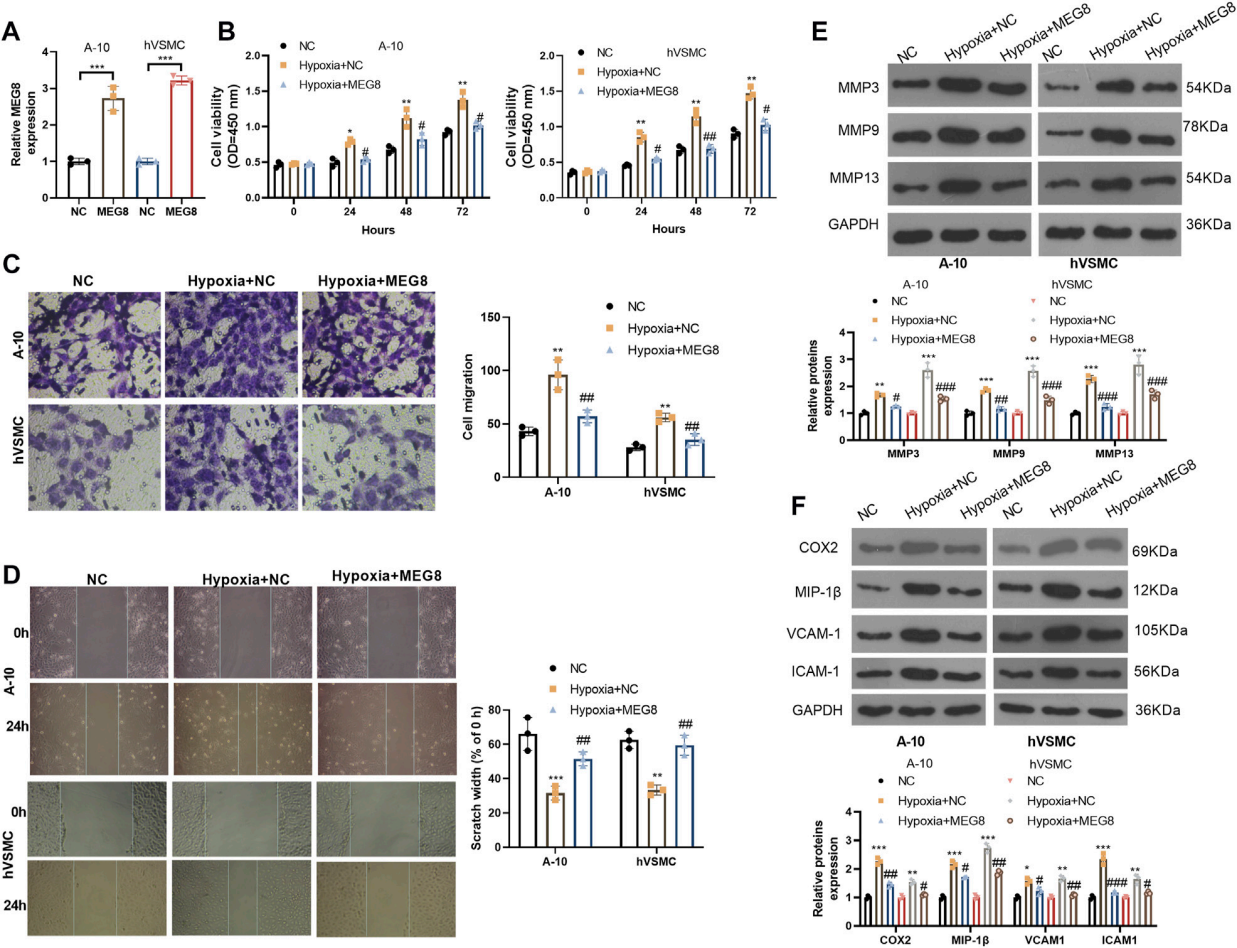
Over-expression of MEG8 attenuated hypoxia-induced VSMC proliferation, inflammation and migration.MEG8 overexpressing plasmids were transfected in A-10 cells or hVSMCs, which were then induced with hypoxia. **(A)** The MEG8 overexpression model was established, and the MEG8 level was detected by qRT-PCR. **(B)** Cell proliferation was verified by the CCK-8 assay. **(C)** Cell migration was determined by the Transwell assay. **(D)** Wound healing assay was employed to analyze cell migration. **(E)** The protein expression of MMP3, MMP9 and MMP13 in A-10 cells or hVSMCs was compared by Western blot. **(F)** The profiles of COX2, MIP-1β, VCAM-1 and ICAM-1 in A-10 cells or hVSMCs were checked by Western blot. *p < 0.05, **p < 0.01, ***p < 0.001 (vs.NC group), #*p* < 0.05, ##*p* < 0.01, ###*p* < 0.001 (vs. hypoxia+NC group). All experiments were repeated three times, and each time was performed in triplicate.

To figure out whether MEG affects the abnormal cellular behaviors of VSMCs, we constructed an overexpression model of MEG8 in A-10 cells and hVSMCs (*p* < 0.05, Figure 2A). Then the two cell lines were treated with hypoxia for 24 h. The CCK-8 assay was employed to check proliferation, and the results exhibited that hypoxia stimulation signally enhanced the proliferation of A-10 cells and hVSMCs (*p* < 0.05, Figure 2B). Overexpressing MEG8 distinctly hindered hypoxia-induced excessive proliferation of VSMCs (*p* < 0.05, Figure 2B). Transwell assay and wound healing assay were conducted for gauging cell migration. As a result, hypoxia facilitated the migration of A-10 cells and hVSMCs. Nevertheless, overexpression of MEG8 significantly reduced VSMC migration (*p* < 0.05, Figures 2C,D). Then, we monitored the expression of MMP3, MMP9, MMP13 and inflammatory proteins (including COX2, MIP-1β, VCAM-1 and ICAM-1) by Western blot, which uncovered that the levels of MMP3, MMP9, MMP13, COX2, MIP-1β, VCAM-1 and ICAM-1 were all enhanced with hypoxia treatment (Figures 2E,F). After MEG8 overexpression, those proteins were all repressed (Figures 2E,F). Thus, overexpression of MEG8 dampened hypoxia-induced VSMC proliferation, migration, extracellular matrix degradation, and inflammatory responses.

### miR-195-5p Up-Regulation Reversed the Protective Effect of Maternally Expressed 8 on Vascular Smooth Muscle Cells

**FIGURE 3:**
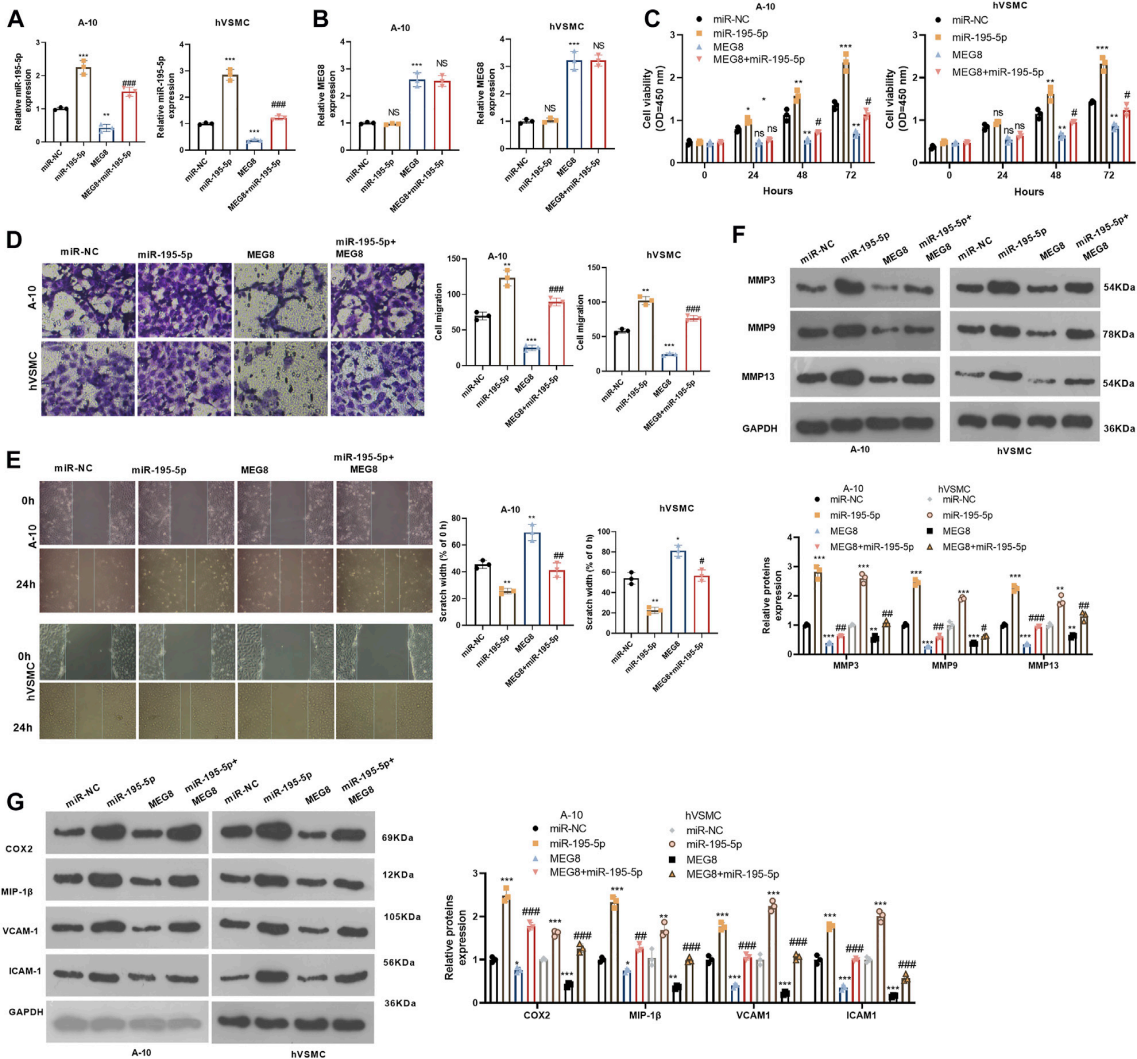
Up-regulating miR-195-5p reversed the protective effect of MEG8 on VSMCs. MEG8 overexpressing plasmids and/or miR-195-5p mimics were transfected in hypoxia-induced A-10 cells or hVSMCs. **(A,B)** The expression of MEG8 and miR-195-5p was monitored by qRT-PCR. C. Cell proliferation was measured by the CCK-8 assay. **(D,E)** Transwell and wound healing assays were used to detect cell migration. **(F,G)** The expression of MMP3, MMP9, MMP13, COX2, MIP-1β, VCAM-1 and ICAM-1 was analyzed by Western blot. NS *p* > 0.05, *p < 0.05, **p < 0.01, ***p < 0.001 (vs. miR-NC group); NS *p* > 0.05, #*p* < 0.05, ##*p* < 0.01, ###*p* < 0.001 (vs. MEG8 group). All experiments were repeated three times, and each time was performed in triplicate.

We further studied the effect of the MEG8/miR-195-5p axis on VSMCs. Hypoxia-induced A-10 cells or hVSMCs were transfected with MEG8 overexpression plasmids with or without miR-195-5p mimics. The expression of miR-195-5p and MEG8 was gauged by qRT-PCR. The results testified that by contrast with the miR-NC group, the miR-195-5p mimics transfection enhanced miR-195-5p levels but had no significant effects on MEG8 (Figures 2A,B). In contrast, MEG8 overexpression reduced the miR-195-5p level (Figures 2A,B). CCK-8 results hinted that cell proliferation was intensified in the miR-195-5p group and was impeded in the MEG8 group. However, the addition of miR-195-5p mimics in the MEG8 group led to a rise in cell proliferation (*p* < 0.05, Figure 3C). In addition, Transwell assay and wound healing assay results showed that in comparison to the miR-NC group, cell migration of the miR-195-5p group was enhanced, while that of the MEG8 group was weakened. Meanwhile, the migration of the MEG8+miR-195-5p group was significantly enhanced compared with that of the MEG8 group (*p* < 0.05, Figures 3D,E). Western blot illustrated that compared with the miR-NC group, the profiles of MMP3, MMP9 and MMP13, as well as the inflammatory proteins (including COX2, MIP-1β, VCAM-1 and ICAM-1) in the miR-195-5p group, were up-lifted, while those in the MEG8 group were curbed. Compared with the MEG8 group, the MMP3, MMP9, MMP13, COX2, MIP-1β, VCAM-1 and ICAM-1 in the MEG8+miR-195-5p group were up-regulated (*p* < 0.05, Figures 3F,G). These data indicated that miR-195-5p enhanced hypoxia-induced VSMC proliferation, migration and inflammation and reversed the inhibitory effect of MEG8 on hypoxia-induced VSMCs.

### Maternally Expressed 8 Targeted miR-195-5p as a Competitive Endogenous

**FIGURE 4:**
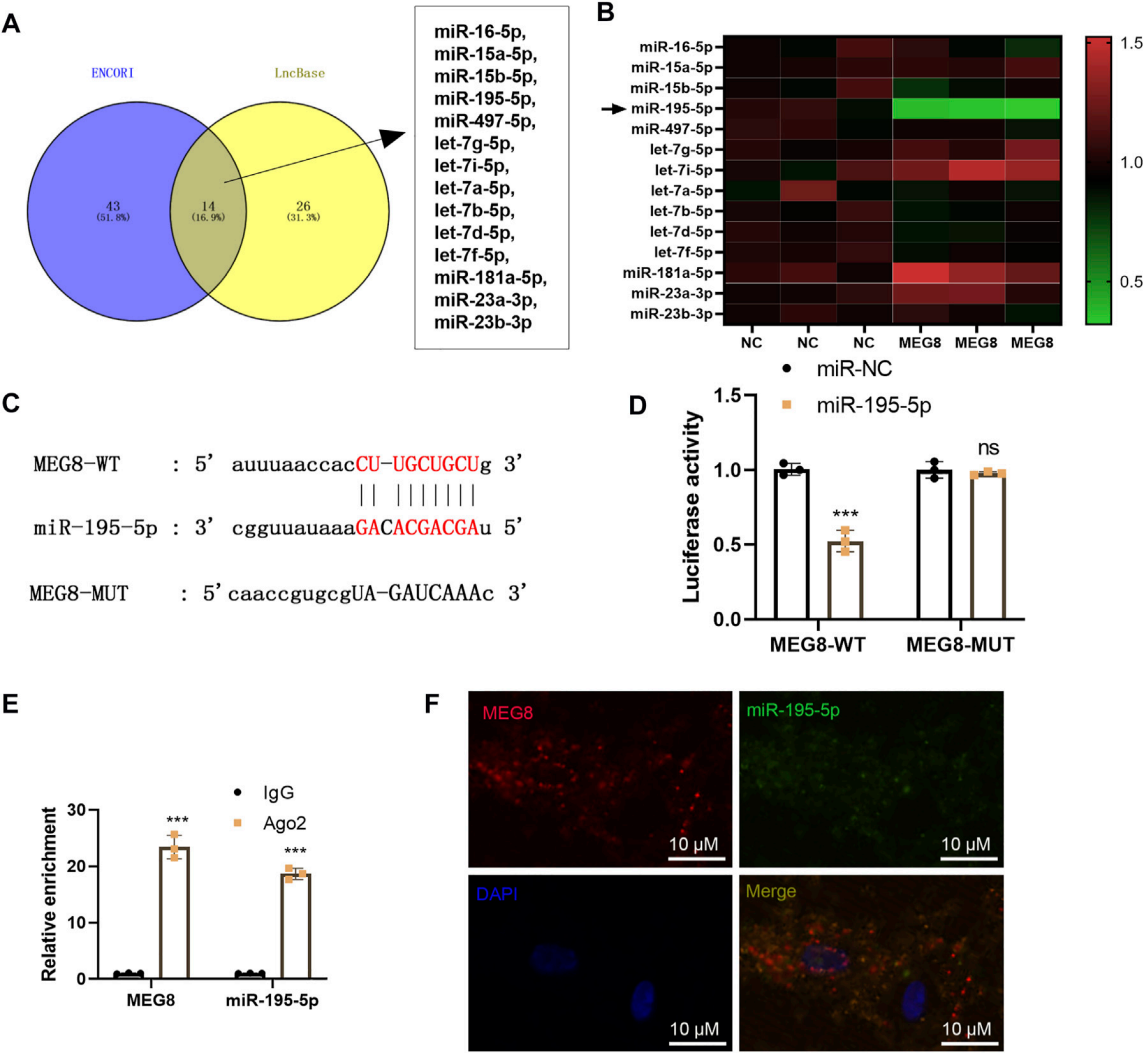
MEG8 targeted miR-195-5p. **(A)** Two online databases, including ENCORI (The Encyclopedia of RNA Interactomes) (http://starbase.sysu.edu.cn/) and LncBase Experimental v.2 (http://carolina.imis.athena-innovation.gr/diana_tools/web/index.php?r=lncbasev2%2Findex) were used for exploring the potential miRNAs as MEG8' targets. **(B)** qRT-PCR was used for detecting hsa-miR-16-5p, hsa-miR-15a-5p, hsa-miR-15b-5p, hsa-miR-195-5p, hsa-miR-497-5p, hsa-let-7g-5p, hsa-let-7i-5p, hsa-let-7a-5p, hsa-let-7b-5p, hsa-let-7d-5p, hsa-let-7f-5p, hsa-miR-181a-5p, hsa-miR-23a-3p, hsa-miR-23b-3p in MEG8-overexpressed A-10 cells. **(C)** The binding sites between MEG8 and miR-195-5p; **(D)** The dual-luciferase reporter assay was implemented to confirm the binding relationship between MEG8 and miR-195-5p. **(E)** RIP verified the binding of MEG8 with miR-195-5p. The enrichment of MEG8 and miR-195-5p was detected by qRT-PCR; **(F)** FISH observed the intracellular binding of miR-195-5p (green) and MEG8 (red). NS *p* > 0.05, ***p < 0.001 (vs. miR-NC group) All experiments were repeated three times, and each time was performed in triplicate.

By searching two online databases, including ENCORI (The Encyclopedia of RNA Interactomes) (http://starbase.sysu.edu.cn/) and LncBase Experimental v.2 (http://carolina.imis.athena-innovation.gr/diana_tools/web/index.php?r=lncbasev2%2Findex), we found that 14 potential miRNAs are MEG8' targets, including hsa-miR-16-5p, hsa-miR-15a-5p, hsa-miR-15b-5p, hsa-miR-195-5p, hsa-miR-497-5p, hsa-let-7g-5p, hsa-let-7i-5p, hsa-let-7a-5p, hsa-let-7b-5p, hsa-let-7d-5p, hsa-let-7f-5p, hsa-miR-181a-5p, hsa-miR-23a-3p, hsa-miR-23b-3p (Figure 4A). We detected those miRNAs in MEG8-overexpressed cells, and found that miR-195-5p was most significantly downregulated after MEG overexpression (Figure 4B). The dual-luciferase reporter assay showed that miR-195-5p mimics declined the luciferase activity of 293T cells transfected with MEG8-WT, but they had no substantial impact on that of 293T cells transfected with MEG8-MUT (Figures 4C,D). RIP results showed that MEG8 and miR-195-5p were enriched in Ago2 immunoprecipitation compared with that in IgG immunoprecipitation (*p* < 0.05, Figure 4E). Besides, FISH confirmed that MEG8 and miR-195-5p were co-localized in the cytoplasm of A-10 cells (Figure 4F). The above results confirmed that MEG8 targeted miR-195-5p.

### miR-195-5p Targeted Reversion Inducing Cysteine Rich Protein With Kazal Motifs and Impeded its Expression

**FIGURE 5:**
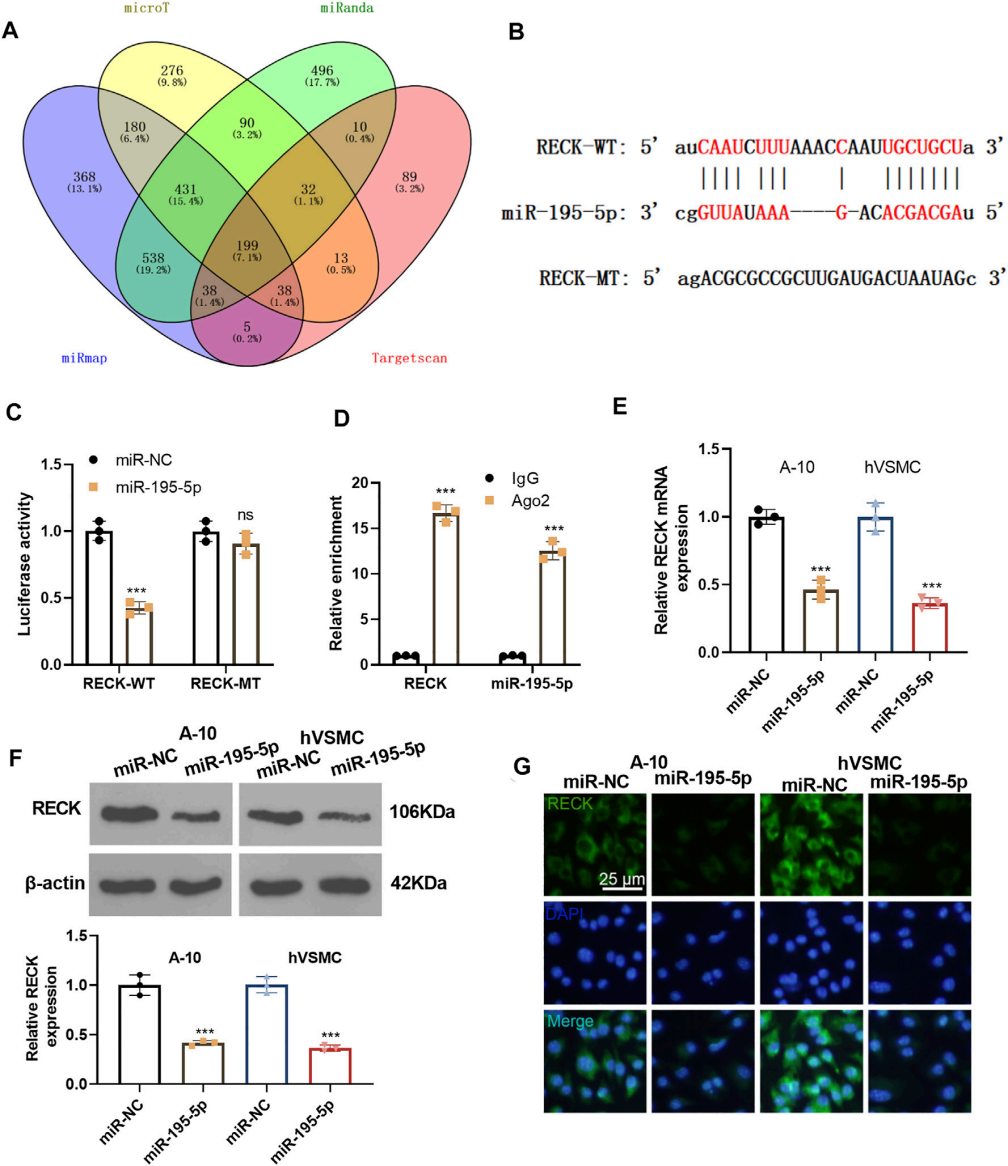
miR-195-5p targeted RECK and inhibited its expression. **(A)** The downstream targets of miR-195-5p were predicted via four databases, including microT, miRanda, miRmap and Targetcan, and the potential targets of miR-195-5p were analyzed through Venn’s diagram. **(B)** The base binding relationship between RECK and miR-195-5p was shown. **(C)** The dual-luciferase reporter assay was adopted to confirm the binding relationship between MEG8 and miR-195-5p. **(D)** RIP verified the binding of MEG8 with miR-195-5p. NS *p* > 0.05, ***p < 0.001 (vs. miR-NC group). **(E,F)** A-10 cells or hVSMCs were transfected with miR-NC or miR-195-5p mimics. The RECK expression was tested by qRT-PCR or Western blot. ***p < 0.001 (vs. miR-NC group). **(G)** The RECK expression in A-10 cells or hVSMCs was gauged by immunofluorescence. All experiments were repeated three times, and each time was performed in triplicate.

We predicted the downstream target of miR-195-5p via four databases, including microT, miRanda, miRmap and Targetcan. Though Venn’s diagram analysis, we discovered that 199 genes were potential targets of miR-195-5p, which include RECK (Figure 5A). The base binding relationship between RECK and miR-195-5p is shown in Figure 5B. The dual-luciferase reporter assay uncovered that the transfection of miR-195-5p mimics declined the luciferase activity of 293T cells transfected with RECK-WT, but it had no inhibitory effect on the luciferase activity of 293T cells transfected with RECK-MT (Figure 5C). RIP assay outcomes showed that compared with the anti-IgG group, the anti-Ago2 group had significantly enriched miR-195-5p and RECK (*p* < 0.05, Figure 5D). The RECK expression was checked by qRT-PCR and Western Blot, which disclosed that the transfection of miR-195-5p mimics hampered the RECK expression (*p* < 0.05, Figures 5E,F). Additionally, cellular immunofluorescence detection displayed that the up-regulation of miR-195-5p restrained the expression of RECK (Figure 5G). These results confirmed that miR-195-5p targeted and curbed RECK expression.

### Overexpressing Reversion Inducing Cysteine Rich Protein With Kazal Motifs Alleviated miR-195-5p-Induced Abnormal Proliferation, Inflammation and Migration of Vascular Smooth Muscle Cells

**FIGURE 6:**
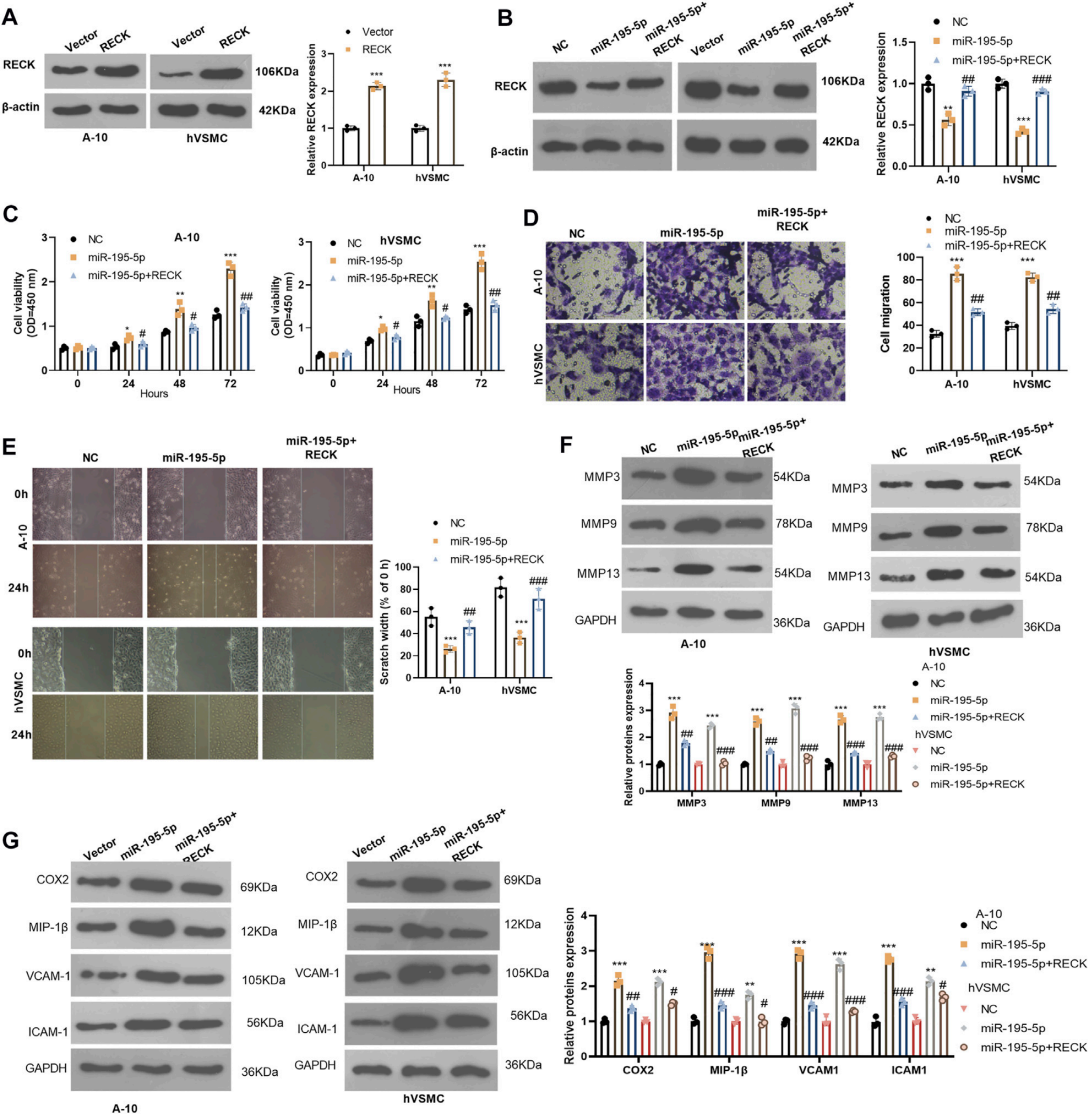
Overexpressing RECK alleviated miR-195-5p-induced abnormal proliferation and migration of VSMCs. **(A)** A-10 cells and hVSMCs were transfected with vectors or RECK overexpression plasmids. The protein level of RECK was gauged by Western blot. **(B)** A-10 cells and hVSMCs were transfected with miR-195-5p mimics and/or RECK overexpression plasmids and then treated with hypoxia. **(B)** Western blot was applied to determine the RECK expression. **(C)** CCK-8 was applied to observe cell proliferation. **(D,E)** Transwell and wound healing assays were performed for evaluating cell migration; **(F,G)** The profiles of MMP3, MMP9, MMP13, COX2, MIP-1β, VCAM-1 and ICAM-1 were analyzed by Western blot. *p < 0.05, **p < 0.01, ***p < 0.001 (vs.Vector group); NS*P* > 0.05, #*p* < 0.05, ##*p* < 0.01 (vs.miR-195-5p group). All experiments were repeated three times, and each time was performed in triplicate.

We probed whether overexpression of RECK interferes with the biological behavior of VSMCs. The RECK overexpression model was constructed in A-10 cells and hVSMCs (compared with the vector group, *p* < 0.05, Figure 6A). We then transfected A-10 cells and hVSMCs with miR-195-5p and/or RECK overexpression plasmids. Western blot showed that compared with that in the NC group, the RECK expression was significantly attenuated in the miR-195-5p group while was up-lifted in the miR-195-5p+RECK group (vs. the miR-195-5p group, *p* < 0.05, Figure 6B). The CCK-8 assay results manifested that the proliferation of the miR-195-5p group was significantly increased compared with that of the NC group, while RECK overexpression decreased cell proliferation (vs. the miR-195-5p group, *p* < 0.05, Figure 6C). Transwell and wound healing assay were used for detecting cell migration, which demonstrated that cell migration was more pronounced in the miR-195-5p group than that in the control group, while the cell migration in the miR-195-5p+RECK group was weaker than that in the miR-195-5p group (*p* < 0.05, Figures 6D,E). Western blot data showed that compared with the NC group, the expression of MMP3, MMP9, MMP13, COX2, MIP-1β, VCAM-1 and ICAM-1 in the miR-195-5p group was uplifted. However, compared with the miR-195-5p group, the profiles of those proteins in the miR-195-5p+RECK group were curbed (*p* < 0.05, Figures 6F,G). In conclusion, overexpression of RECK reduced the abnormal biological behaviors of VSMCs induced by miR-195-5p under hypoxia.

### Inhibiting Hypoxia Inducible Factor 1 Subunit Alpha Improved Hypoxia-Induced Proliferation and Migration of Vascular Smooth Muscle Cells by Enhancing Maternally Expressed 8 and Reducing miR-195-5p

**FIGURE 7:**
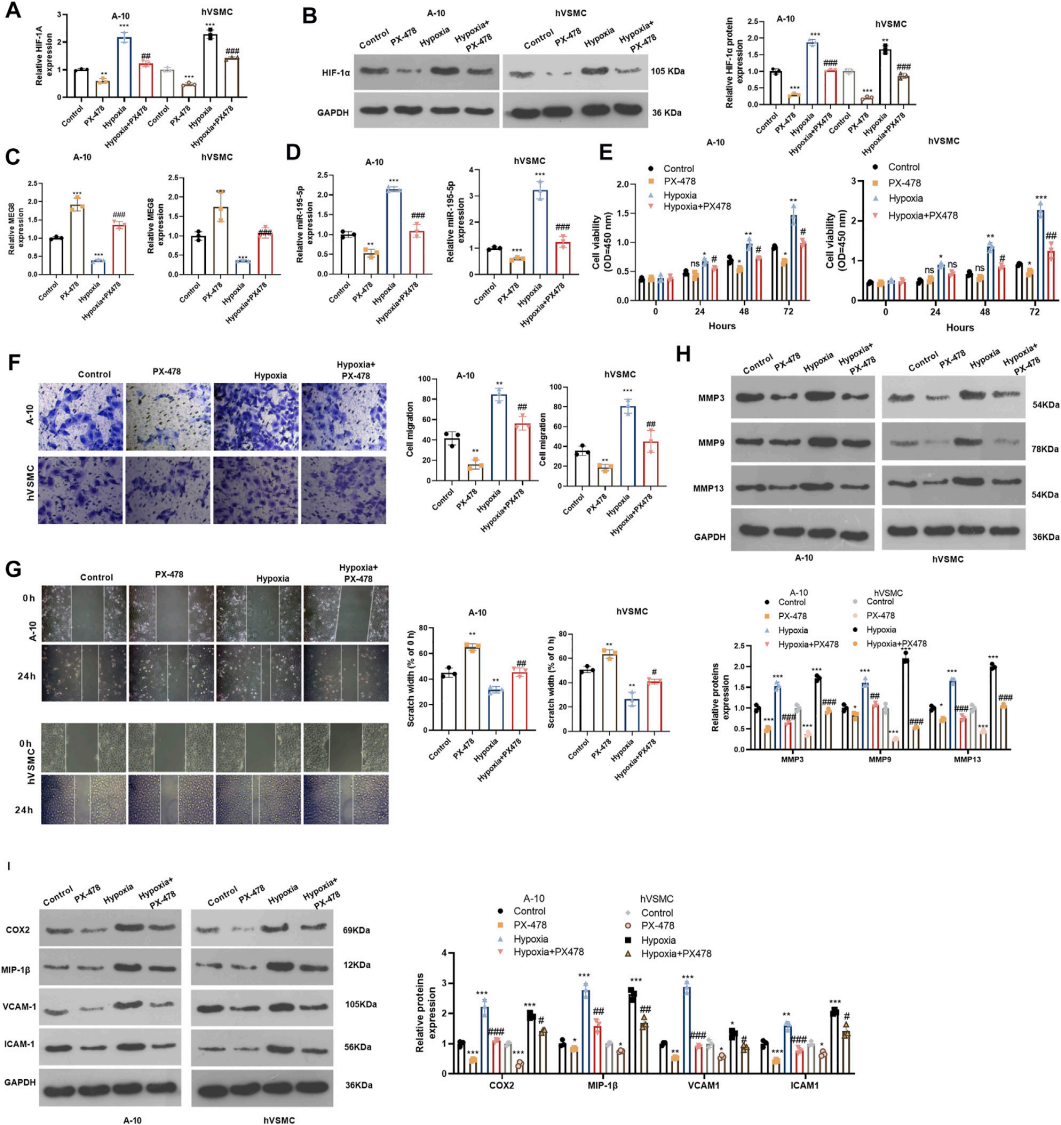
Inhibiting HIF-1α alleviated hypoxia-induced abnormal proliferation and migration of VSMCs and enhanced MEG8 expression. A-10 cells and hVSMCs were stimulated by hypoxia, and treated with, or without HIF-1α inhibitor PX478 (25 μM). **(A,B)** The mRNA and protein level of HIF-1α were tested by qRT-PCR **(A)** and western blot **(B)**, respectively. **(C,D)** The expression of MEG8 and miR-195-5p was monitored by qRT-PCR. **(E)** Cell proliferation was measured by the CCK-8 assay. **(F,G)** Transwell and wound healing assays were used to detect cell migration. **(H,I)** The expression of MMP3, MMP9, MMP13, COX2, MIP-1β, VCAM-1 and ICAM-1 was analyzed by Western blot. NS *p* > 0.05, *p < 0.05, **p < 0.01, ***p < 0.001 (vs. Control group); NS *p* > 0.05, #*p* < 0.05, ##*p* < 0.01, ###*p* < 0.001 (vs. Hypoxia group). All experiments were repeated three times, and each time was performed in triplicate.

For confirming HIF-1α’s role in hypoxia-induced abnormal proliferation and migration of VSMCs, we dealt A-10 cells and hVSMCs under hypoxia treatment with HIF-1α inhibitor PX478 (25 μM). By examining HIF-1α mRNA and protein alteration, it was found that PX478 inhibited HIF-1α level, which was significantly increased in A-10 cells and hVSMCs under hypoxia (Figures7A,B). Next, we tested he expression of MEG8 and miR-195-5p. The qRT-PCR result indicated that PX478 enhanced MEG8, whereas inhibited miR-195-5p level (*p* < 0.05 compared with Control or Hypoxia group, Figures 7C,D). Cell proliferation was measured by the CCK-8 assay, and cell migration was evaluated by Transwell and wound healing assays. The data revealed that the HIF-1α inhibitor PX478 had a slight role in inhibiting cell proliferation and migration under normal incubation (compared with Control group, Figures 7E–G). Under hypoxia stimulation, PX478 treatment remarkably repressed cell proliferation and migration (compared with hypoxia group, Figures 7E–G). Furthermore, the expression of MMP3, MMP9, MMP13, COX2, MIP-1β, VCAM-1 and ICAM-1 was analyzed by Western blot. As shown by the data, PX478 treatment inhibited those proteins both in the normal and hypoxia environment (Figures 7H,I). Taken together, inhibiting HIF-1α reversed hypoxia-mediated proliferation, migration and inflammation by upregulating MEG8.

## Discussion

**FIGURE 8:**
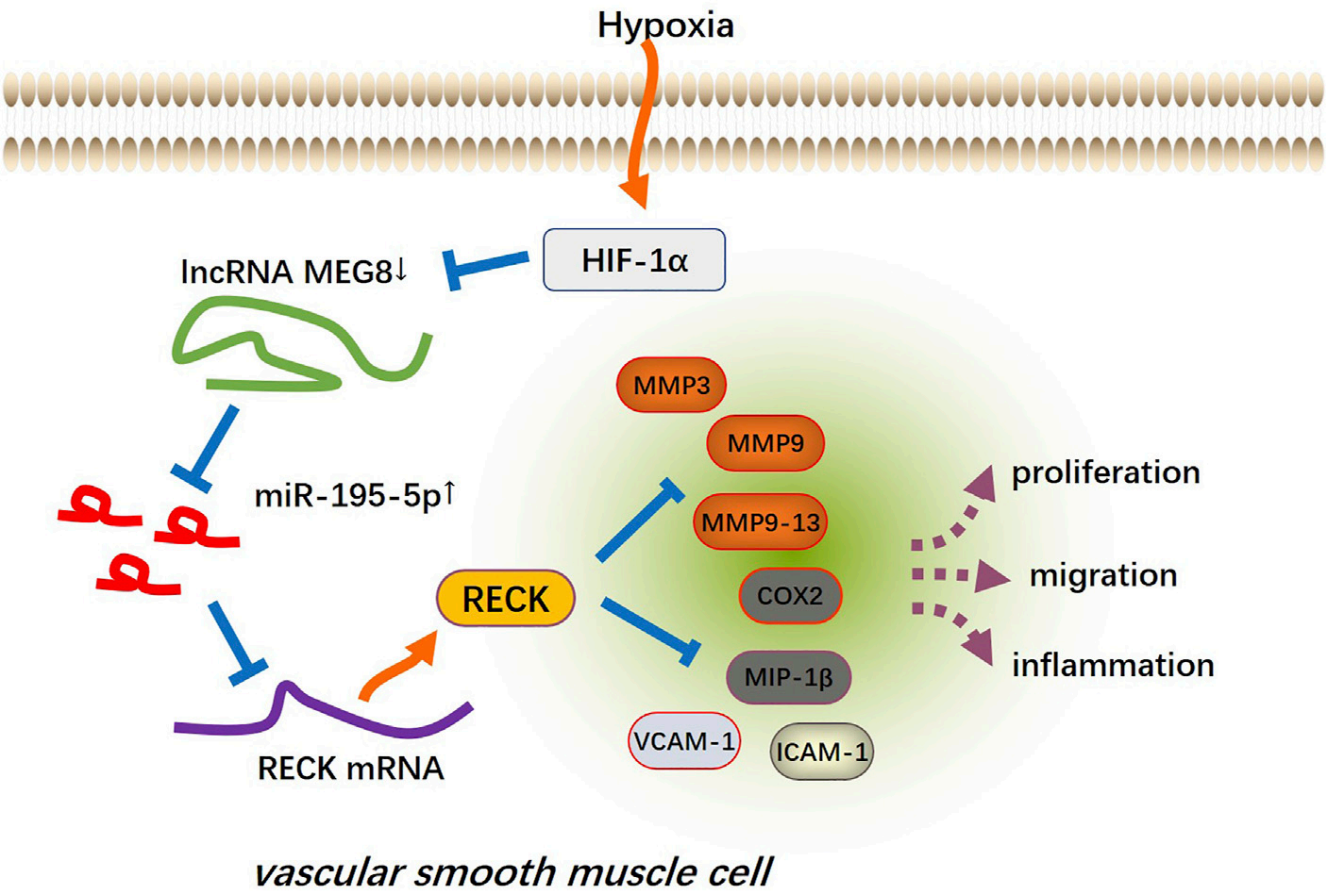
The mechanism’s diagram. The up-regulation of MEG8 in hypoxia-induced VSMCs uplifted the expression of RECK by targeting and inhibiting miR-195-5p, thus reducing excessive cell proliferation, inflammation and migration.

Cardiovascular and cerebrovascular diseases are the main health problems affecting human health, and the dysfunction of VSMCs is involved in the pathogenesis of vascular diseases. VSMCs have strong plasticity, in which proliferation, contraction, ECM synthesis and other functions can be performed. Under normal conditions, the growth rate and synthesis activity of VSMCs are low enough to maintain vascular tension and regulate blood pressure. When pathological stimulation occurs, VSMCs produce abnormal proliferation to repair the vascular injury, thus inducing the formation of new intima and causing local vascular lumen stenosis (Lacolley et al., 2012; Beermann et al., 2018). Presently, we found that MEG8 was downregulated in hypoxia-treated VSMCs, and MEG8 regulated the miR-195-5p/RECK axis to reduce the abnormal proliferation, inflammation and migration of VSMCs (Figure 8).

LncRNAs were previously ignored due to their lack of protein-coding function. However, with the deepening of the research, it has been confirmed that lncRNAs are crucial to biological development and disease occurrence, including mediating cell proliferation, differentiation, apoptosis, autophagy, drug resistance, etc. (Bhat et al., 2020; Huang et al., 2021; Wu et al., 2021). Of special note, lncRNAs also affect the proliferation and migration of VSMCs and then participate in vascular diseases. Sun et al. claimed that inhibition of lncRNA H19 reduced VSMC proliferation and increased apoptosis, which helped slow down atherosclerosis (Sun et al., 2020). Zheng et al. found that overexpression of SNHG7 targeted and inhibited miR-1306-5p/Sirtuin7, reducing excessive proliferation, migration and invasion of VSMCs induced by oxidized low-density lipoprotein and alleviating atherosclerosis (Zheng et al., 2021). Some scholars have also stated that LINC00961 is a potential target for the treatment of atherosclerosis. Overexpressing LINC00961 reduces the proliferation of VSMCs induced by hypoxia and C-reactive protein and promotes apoptosis by inhibiting miR-367 (Wu et al., 2019). Wang et al. showed that knocking down MEG3 in VSMCs induced by oxidization-low density lipoprotein boosts the proliferation of VSMCs and inhibits apoptosis by regulating the miR-361-5p/ABCA1 axis (Wang et al., 2019). MEG8, a newly discovered lncRNA, is implicated in various diseases. Buccarelli et al. showed that MEG8 plays a tumor-suppressive role in glioblastoma, reduces the viability, migration and EMT of tumor cells, and hinders tumor growth *in vivo* (Buccarelli et al., 2020). Some scholars have also found that MEG8 is up-regulated in the plasma of patients with gestational diabetes, which is helpful to predict the degree of kidney injury (Zhang et al., 2021). In addition, MEG8 is highly expressed in human spontaneous abortion villi, and overexpressing MEG8 induces abortion and trophoblast cell dysfunction (Sheng et al., 2019). More importantly, MEG8 targets the miR-181a/peroxisome proliferator-activated receptors-alpha (PPARα) axis to repress the proliferation and migration of VSMCs and heighten apoptosis (Zhang et al., 2019).

Our study showed that MEG8 was lowly expressed in the carotid artery of rats with balloon injury and the aortic smooth muscle cells of rats treated with hypoxia, and MEG8 overexpression abated the excessive proliferation and migration of hypoxia-induced VSMCs. Previous studies have confirmed that targeting vascular remodeling to treat pulmonary arterial hypertension (Thompson and Lawrie, 2017). LncRNA has been found exerting possible role in treating PAH. For instance, Zehendner et al. (2020) have shown that knockdown of lncRNA TYKRIL in precision-cut lung slices decreased the vascular remodeling and proliferating (Zehendner et al., 2020). Su et al. (2018) suggested that lncRNA H19 knockout protected mice from pulmonary artery remodeling and Pulmonary arterial hypertension (PAH) following monocrotaline (MCT) treatment (Su et al., 2018). Basing on our *in-vitro* finding, we also believed that targeting might be helpful in treating PAH. Anyhow, this needs to be confirmed in *in-vivo* experiments.

As a family of zinc-dependent endopeptidases, matrix metalloproteinases (MMPs) are involved in the degradation of various proteins in the ECM, thereby affect the migration and proliferation of VSMCs (Cui et al., 2017; Wang and Khalil, 2018). Moreover, over-produced inflammatory mediators have essential effects on VSMCs. For example, on homocysteine (Hcy)-induced rat cerebral artery VSMCs, MMP-14, the Toll-like receptor (TLR4)/nuclear transcription factor-κB (NF-κB) pathway and the inflammatory cytokines including Interleukin 6 (IL-6) and Tumor necrosis factor-alpha (TNF-alpha) are augmented (Ma et al., 2020). In addition, AngII boosts COX-2 and tenascin-C expression and cell migration in VSMCs induced by IL-1β (Aguado et al., 2015). Hence, those MMPs and inflammatory mediators were vital in controlling the VSMC migration and remodeling. Here, we found that hypoxia promoted MMP3, MMP9, MMP13, COX2, MIP-1β, VCAM-1 and ICAM-1 expression in VSMCs. However, overexpression of MEG8 down-regulated those proteins, suggesting that MEG8 reduced hypoxia-induced VSMC migration through repressing inflammation.

miRNAs are critical regulators in biological processes. In addition to the extensive involvement in the progression of malignant tumors, miRNAs also interfere with the functions of endothelial cells, VSMCs and macrophages, affecting atherosclerosis and modulating lipid metabolism (Aryal et al., 2014). Some scholars have reported that the bone morphogenetic protein (BMP) pathway dampens the dedicator of cytokinesis 4 (DOCK4) profile by up-regulating miR-101, thereby reducing the proliferation and migration of VSMCs (Park and Kang, 2020). Also, miR-637 targets IGF-2 to decline the proliferation and migration and promote apoptosis of VSMCs, contributing to the treatment of atherosclerosis (Yang et al., 2020). However, miR-145 is lowly expressed in carotid arteries of intimal hyperplasia mice, and overexpression of miR-145 hampers the proliferation and migration of VSMCs by abating autophagy, which can be used to prevent cardiovascular diseases (Wang et al., 2020b). However, other studies have shown that GAS5 targets miR-21 to alleviate the proliferation and migration of VSMCs induced by platelet-derived growth factor-BB (PDGF-BB) (Liu et al., 2019). These studies suggest that miRNAs mediate the proliferation, migration and apoptosis of VSMCs, and are potential targets for the treatment of atherosclerosis and other vascular diseases. In recent years, emerging studies on miR-195-5p have been conducted. miR-195-5p targets EZH2 to inhibit gestational diabetes mellitus-induced human umbilical vein endothelial cells’ proliferation and promote their apoptosis (Liao et al., 2020). More importantly, miR-195-5p secreted by pulmonary microvascular endothelial cells induces the proliferation and migration of lung smooth muscle cells (Zeng et al., 2018). Through experiments, we found that miR-195-5p was highly expressed in the injured carotid artery and hypoxia-induced VSMCs in rats, and miR-195-5p was the downstream target of MEG8. The protective effect of MEG8 on VSMCs was restrained as the proliferation, inflammation and migration of cells transfected with miR-195-5p mimics were significantly enhanced.

RECK is an emerging transcriptional suppressor gene that is widely expressed in the human body in recent years and can inhibit the activation and secretion of MMPs (Oh et al., 2001). According to reports, RECK can reduce the invasion and migration of malignant tumors by abating MMP-9 secretion (Takahashi et al., 1998). Jian et al. showed that miR-30b-3p was overexpressed in glioma, and RECK could be up-regulated by inhibiting miR-30b-3p, thereby reducing the proliferation, migration and invasion of tumor cells and declining the protein levels of MMP-2 and MMP-9 (Jian et al., 2019). STAT3 curbs the RECK expression and increases the activity of MMP-2 and MMP-9 in lung cancer cells by up-regulation of miR-92a, promoting the malignant biological behavior of cells (Lin et al., 2013). More importantly, MMP degrades both ECM and non-matrix substrates, which are important for vascular remodeling. Mummidi et al. found that RECK reduced VSMCs of the human aorta by inhibiting the IL-17A/TRAF3IP2 signal transduction and MMP-13 release, thus leading to the thickening of the new intima in tissue proliferative vascular diseases (Mummidi et al., 2019). In addition, Higashi et al. also demonstrated that minocycline inhibited miR-221, and miR-222 up-regulated RECK, which reduced PDGF-BB-induced VSMC proliferation and migration in the human aorta (Higashi et al., 2019). Fortunately, we found through experiments that miR-195-5p targeted and inhibited RECK, and overexpression of RECK partially reversed the proliferation and migration of miR-195-5p-mediated and hypoxia-induced VSMCs and the protein expression of MMP3, 9, 13 and inflammatory responses.

Hypoxia-inducible factor-1α (HIF-1α) is an alpha subunit of transcription factor HIF-1, which has been regarded as a master regulator of cellular and systemic homeostatic response to hypoxia (Choudhry and Harris, 2018). Many genes, which are involved in energy metabolism (Hasegawa et al., 2020), angiogenesis (Semenza, 2014), apoptosis (Carmeliet et al., 1998), inflammation (Fu et al., 2020), oxidative stress (Chu et al., 2019), and so on, are increasingly transcribed by HIF-1. HIF-1α upregulation induced by hypoxic stimulation is involved in vascular remodeling (Luo et al., 2019). Hif1a knockout in VSMC led to restrained M1 macrophages into the vessel-induced by Ang II (Qi et al., 2019). Moreover, HIF-1α controls the expression of lncRNA and miRNA at the transcriptional level (Chen et al., 2019; Xu et al., 2019). Here, we tested MEG8 level change after HIF-1α inhibition. It was found that HIF-1α inhibitor PX-478 significantly enhanced MEG8 level, whereas repressed miR-195-5p expression. Moreover, suppressing HIF-1α reversed hypoxia-induced dysfunction of VSMC. Hence, we believed that MEG8 is potentially regulated by HIF-1α in hypoxia-stimulated VSMC.

In conclusion, overexpression of MEG8 alleviated hypoxia-induced VSMC proliferation, inflammation and migration by targeting the miR-195-5p/RECK axis (Figure 8). HIF-1α plays a potential role in mediating MEG8-miR-195-5p axis. This article provides a new direction for MEG8 to treat vascular diseases such as atherosclerosis. However, this study was only tested in animal models and cells and lacked relevant clinical samples, and the regulatory mechanism of the HIF-1α-MEG8-miR-195-5p-RECK axis in hypoxia-induced vascular remodeling needs to be investigated.

## Data Availability

The original contributions presented in the study are included in the article/Supplementary Material, further inquiries can be directed to the corresponding author.

## Glossary

AMPK: AMP-activated protein kinase
ANRIL: antisense non-coding RNA in the INK4 locus
ATCC: American Type Culture Collection
BCA: Bicinchoninic Acid
CASC9: cancer susceptibility candidate 9
CCK-8: cell counting kit-8
ceRNA: competitive endogenous
COX2: cytochrome c oxidase subunit II
DAPI: 4',6-diamidino-2-phenylindole
DMEM: Dulbecco’s modified Eagle’s medium
ECM: extracellular matrix
EZH2: enhancer of zeste homolog 2
FBS: fetal bovine serum
FISH: Fluorescence in situ hybridization
FNDC5: fibronectin type III domain containing 5
H&E: hematoxylin-eosin
HIF-1α: hypoxia inducible factor 1 subunit alpha
ICAM-1: intercellular adhesion molecule 1
IL-6: Interleukin 6
lncRNA: long non-coding RNA
LPS: lipopolysaccharide
MEG8: maternally expressed 8
MAPK: mitogen-activated protein kinase
MIP-1β: macrophage inflammatory protein-1β
MMP: matrix metalloproteinase
NF-κB: nuclear transcription factor-κB
OD: optical density
PDGF: platelet-derived growth factor
PPARα: peroxisome proliferator-activated receptors-alpha
qRT-PCR: quantitative reverse transcription-polymerase chain reaction
RECK: reversion inducing cysteine rich protein with kazal motifs
RIP: RNA immunoprecipitation
SD: Sprague-Dawley
SNHG14: small nucleolar RNA host gene 14
TLR4: Toll-like receptor 4
TNF-alpha: Tumor necrosis factor-alpha
VCAM-1: vascular cell adhesion molecule 1
VEGFA: vascular endothelial growth factor A
VSMCs: vascular smooth muscle cells
YAP1: Yes-associated protein 1
