# Supplementary figures and images for: Long Non-Coding RNA MEG8 Suppresses Hypoxia-Induced Excessive Proliferation, Migration and Inflammation of Vascular Smooth Muscle Cells by Regulation of the miR-195-5p/RECK Axis
Source: Front Mol Biosci. 2021 Nov 1;8:697273. doi: 10.3389/fmolb.2021.697273 (PMC8592128; doi:10.3389/fmolb.2021.697273)

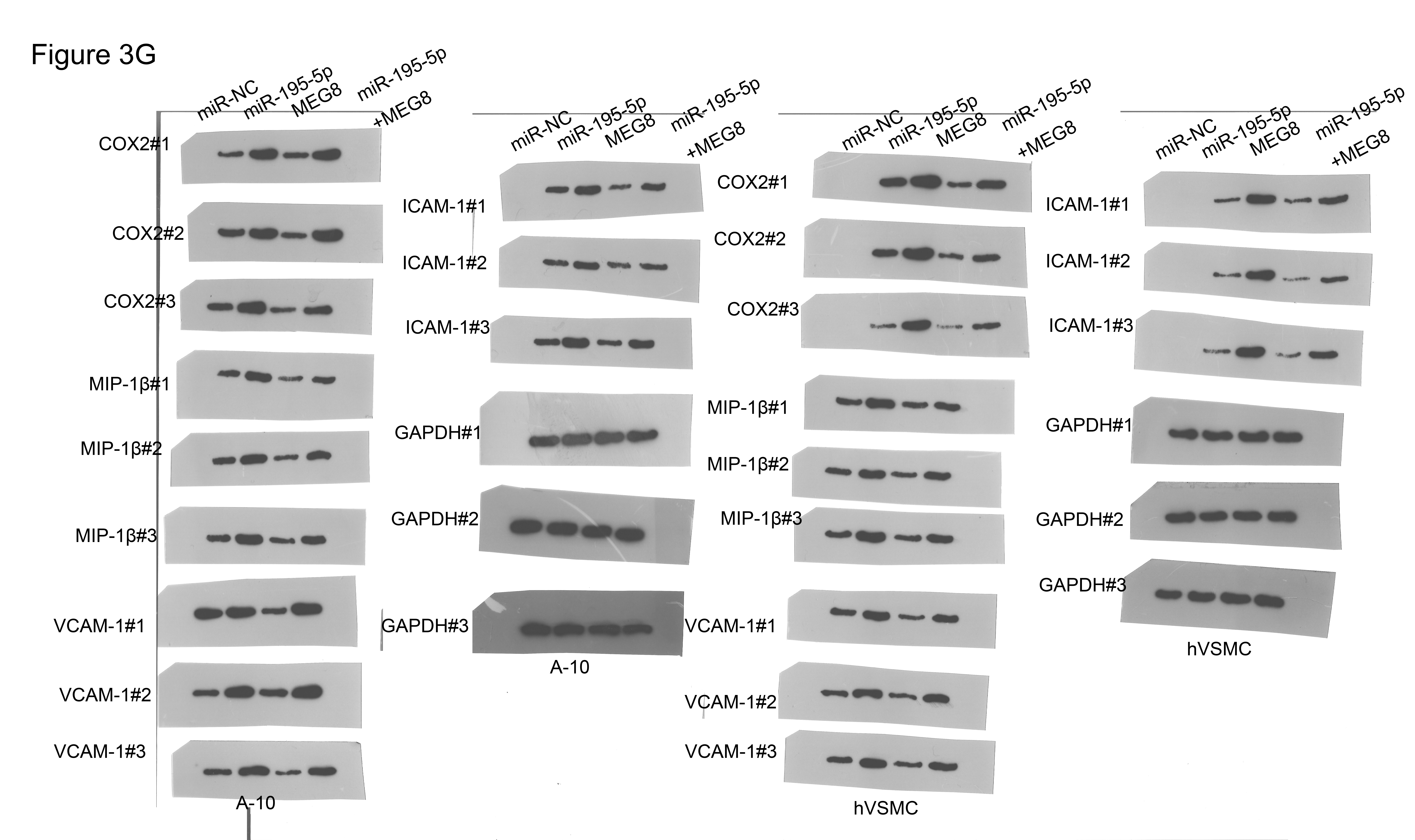

Supplement: Supplementary file 1 [file Image3.JPEG]

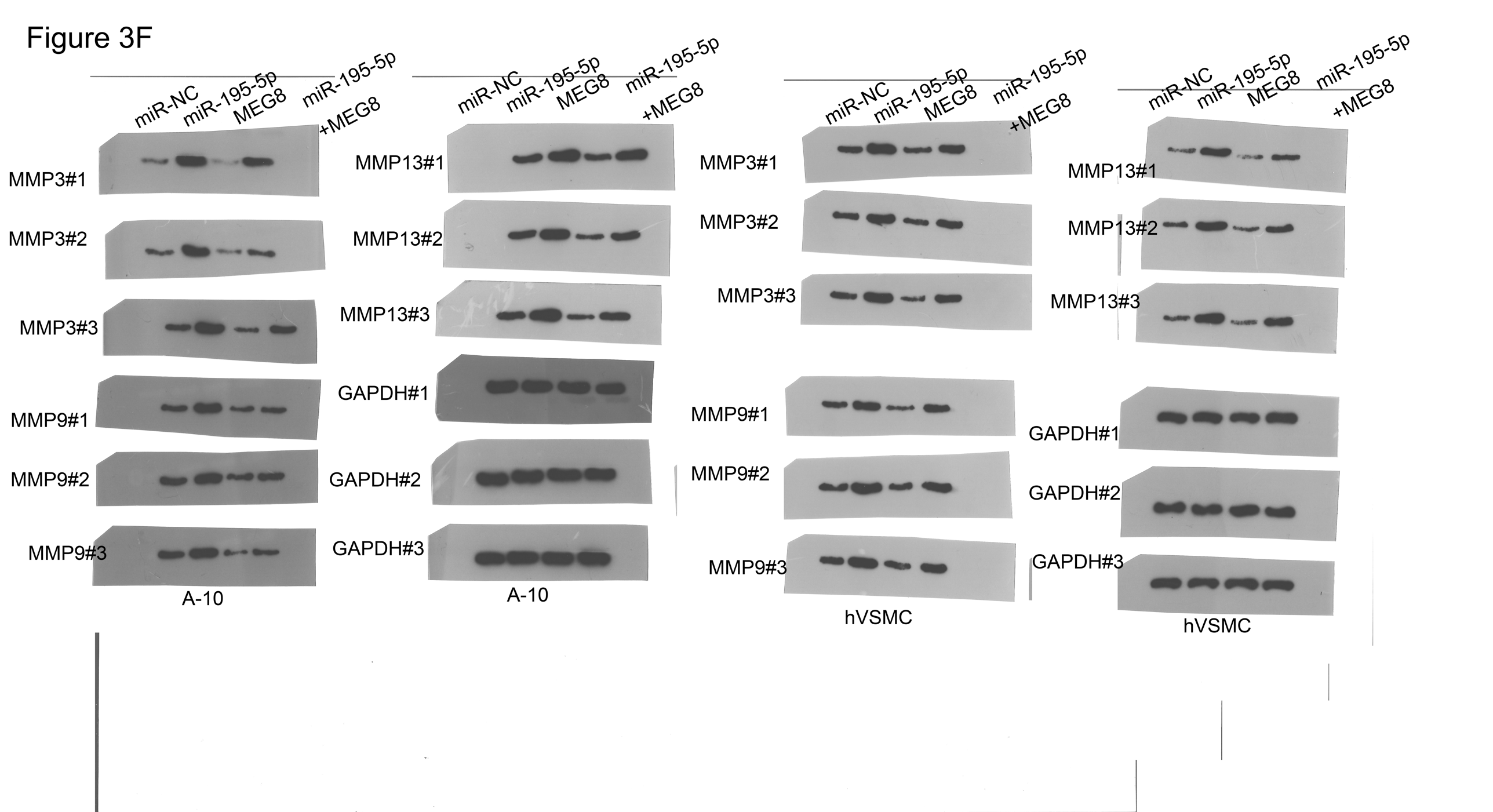

Supplement: Supplementary file 4 [file Image2.JPEG]
